# Supplementary material for: Analysis of PPARγ Signaling Activity in Psoriasis
Source: Int J Mol Sci. 2021 Aug 10;22(16):8603. doi: 10.3390/ijms22168603 (PMC8395241; doi:10.3390/ijms22168603)
Supplement: Supplementary file 1 [file ijms-22-08603-s001.zip › Supplemental materials_Analysis of PPARg signaling activity in psoriasis/Pathway models/Models images and html files/Model of PPARG related pathways in psoriasis (short version)/13381.html]

RIPK2


# Protein RIPK2

|  |  |
| --- | --- |
| URN | urn:agi-llid:8767 |
| Total Entities | 0 |
| Connectivity | 956 |
| Name | RIPK2 |
| Description | receptor interacting serine/threonine kinase 2 |
| Notes | This gene encodes a member of the receptor-interacting protein (RIP) family of serine/threonine protein kinases. The encoded protein contains a C-terminal caspase activation and recruitment domain (CARD), and is a component of signaling complexes in both the innate and adaptive immune pathways. It is a potent activator of NF-kappaB and inducer of apoptosis in response to various stimuli. [provided by RefSeq, Jul 2008] |
| Primary Cell Localization | Cytoplasm |
| Class | Protein kinase |
| ObjectType | Protein |

---

|  |  |
| --- | --- |
| Pathway | NOD-like Receptors |
|  | Crohn's Disease |
|  | Paneth-Cell Function in Crohn's Disease |
|  | Macrophage Dysfunction |
|  | Dendritic Cell Dysfunction in Crohn's disease |
|  | CagA Phosphorylation Independent Signaling |
|  | Dendritic Cell Function in Ulcerative Colitis |
|  | Blau Syndrome |
|  | Proteins Involved in Huntington Disease |
|  | HPV E6 and E7 Induced Disruption of TNFR/NF-kB Signaling in Keratinocytes |
|  | Model of PPARG signaling in psoriasis |
|  | Model of PPARG related pathways in psoriasis (short version) |
|  | 1\_Insufficient activation of immune response in the middle ear epithelium cells in otitis media |
|  | 3\_Role of alveolar macrophages in pulmonary tissue destruction in COPD |
|  | 1\_Th2 cell response in asthma |
|  | 2\_Paneth cell dysfunction in Crohn's disease |
|  | 1\_Defects in response to pathogens in the gut promotes inflammation in Crohn's disease |
|  | 3\_Autophagy dysfunction in Crohn's disease |
|  | 2\_Intestine epithelial cells drive immunological responses in ulcerative colitis |
|  | 1\_Antigen-presenting cells promote islet dysfunction and immune system activation in T1D |
|  | mapk1 protein modificators |

---

|  |  |
| --- | --- |
| MedScan ID | 8767 |

---

|  |  |
| --- | --- |
| LocusLink ID | 8767 |
|  | 192656 |
|  | 362491 |

---

|  |  |
| --- | --- |
| Alias | CARDIAK,CCK,RICK,RIP |
|  | RIKEN cDNA 2210420D18 gene |
|  | receptor interacting protein 2 |
|  | receptor interactive protein II |
|  | receptor-interactive serine-threonine kinase 2 |
|  | RIP-like-interacting CLARP kinase |
|  | DNA segment, Chr 4, Brigham & Women's Genetics 0615 expressed |
|  | RP23-378L22.1 |
|  | CARD-containing IL-1 beta ICE-kinase |
|  | receptor (TNFRSF)-interactive serine-threonine kinase 2 |
|  | CARD-containing interleukin-1 beta-converting enzyme (ICE)-associated kinase |
|  | tyrosine-protein kinase RIPK2 |
|  | CARD3 |
|  | 2210420D18Rik |
|  | growth-inhibiting gene 30 |
|  | GIG30 |
|  | receptor-interacting serine-threonine kinase 2 |
|  | RICK |
|  | OTTMUSP00000004983 |
|  | CARD-carrying kinase |
|  | CARDIAKs |
|  | WUGSC:H\_RG437L15.1 |
|  | receptor-interacting protein (RIP)-like interacting caspase-like apoptosis regulatory protein (CLARP) kinase |
|  | D4Bwg0615e |
|  | CARDIAK |
|  | receptor (TNFRSF)-interacting serine-threonine kinase II |
|  | receptor-interactive serine-threonine kinase II |
|  | (CARD)-containing serine/threonine kinase Rip2 |
|  | receptor (TNFRSF)-interacting serine-threonine kinase 2 |
|  | receptor interacting protein II |
|  | receptor (TNFRSF)-interactive serine-threonine kinase II |
|  | receptor interactive protein 2 |
|  | CARD-containing interleukin-1 beta-converting enzyme-associated kinase |
|  | 2210420D18 |
|  | RIP serine threonine kinase 2 |
|  | RIPK2 |
|  | RIP2 |
|  | receptor-interacting serine-threonine kinase II |
|  | CCK |
|  | RIP-2 |
|  | receptor-interacting protein 2 |
|  | receptor-interacting serine/threonine-protein kinase 2 |

---

|  |  |
| --- | --- |
| EC Number | 2.7.10.2 |
|  | 2.7.11.1 |

---

|  |  |
| --- | --- |
| GO ID | 0005524 |
|  | 0050700 |
|  | 0004706 |
|  | 0030274 |
|  | 0089720 |
|  | 0004715 |
|  | 0042803 |
|  | 0004674 |
|  | 0005102 |
|  | 0007249 |
|  | 0007254 |
|  | 0000165 |
|  | 0042098 |
|  | 0050852 |
|  | 0007256 |
|  | 0007257 |
|  | 0000187 |
|  | 0097202 |
|  | 0002250 |
|  | 0006915 |
|  | 0071223 |
|  | 0071225 |
|  | 0071224 |
|  | 0050830 |
|  | 0006954 |
|  | 0045087 |
|  | 0070498 |
|  | 0031663 |
|  | 0043066 |
|  | 0070427 |
|  | 0070431 |
|  | 0070423 |
|  | 0018108 |
|  | 0070374 |
|  | 0043123 |
|  | 0051092 |
|  | 0045627 |
|  | 0046641 |
|  | 0043065 |
|  | 0010942 |
|  | 0032722 |
|  | 0001961 |
|  | 0033091 |
|  | 0032727 |
|  | 0032728 |
|  | 0032729 |
|  | 0050718 |
|  | 0032735 |
|  | 0032743 |
|  | 0032755 |
|  | 0033138 |
|  | 0010800 |
|  | 0050731 |
|  | 0032092 |
|  | 0031398 |
|  | 0045944 |
|  | 0032760 |
|  | 1904417 |
|  | 0043330 |
|  | 0070671 |
|  | 0070673 |
|  | 0007165 |
|  | 0034134 |
|  | 0034142 |
|  | 0005737 |
|  | 0005856 |
|  | 0005829 |
|  | 0032991 |
|  | 0031982 |
|  | 0016301 |
|  | 0000166 |
|  | 0004672 |
|  | 0016740 |
|  | 0071222 |
|  | 0002376 |
|  | 0016310 |
|  | 0046330 |
|  | 0002827 |
|  | 0032874 |
|  | 0006468 |
|  | 0042981 |
|  | 0043281 |
|  | 0070555 |
|  | 0004871 |
|  | 0043234 |
|  | 0002755 |
|  | 0002756 |
|  | 0035666 |
|  | 0008063 |
|  | 0048011 |
|  | 0035872 |
|  | 0051403 |
|  | 0034130 |
|  | 0034138 |
|  | 0002224 |
|  | 0016772 |
|  | 0005622 |
|  | 0006916 |
|  | 0004872 |
|  | 0034166 |
|  | 0034146 |
|  | 0034162 |
|  | 0038123 |
|  | 0038124 |

---

|  |  |
| --- | --- |
| KEGG ID | hsa:8767 |
|  | mmu:192656 |
|  | rno:362491 |

---

|  |  |
| --- | --- |
| Organism | Homo sapiens {Organism urn:agi-taxid:9606} |
|  | Mus musculus {Organism urn:agi-taxid:10090} |
|  | Rattus norvegicus {Organism urn:agi-taxid:10116} |
|  | Homo sapiens |
|  | Mus musculus |
|  | Rattus norvegicus |

---

|  |  |
| --- | --- |
| Mouse chromosome position | 4 6.7 cM |
|  | 4 5.4 cM |

---

|  |  |
| --- | --- |
| OMIM ID | 603455 |

---

|  |  |
| --- | --- |
| Rat chromosome position | 5q13 |

---

|  |  |
| --- | --- |
| Hugo ID | 10020 |
|  | HGNC:10020 |

---

|  |  |
| --- | --- |
| Human chromosome position | 8q21.3 |
|  | 8q21 |

---

|  |  |
| --- | --- |
| Swiss-Prot Accession | A0A0S2Z4Z8 |
|  | O43353 |
|  | O43353.2 |
|  | P58801 |
|  | Q547H1 |
|  | P58801.1 |
|  | G3V783 |
|  | B7Z748 |
|  | Q6UWF0 |
|  | Q2TU65 |
|  | Q6Q322 |
|  | Q3B7U0 |

---

|  |  |
| --- | --- |
| GenBank ID | NC\_000008 |
|  | XM\_005251092 |
|  | XP\_005251149 |
|  | NM\_003821 |
|  | NP\_003812 |
|  | XM\_011517357 |
|  | XP\_011515659 |
|  | NG\_033016 |
|  | AC004003 |
|  | AAC24561 |
|  | AF117829 |
|  | CH471060 |
|  | EAW91655 |
|  | EAW91656 |
|  | KC877257 |
|  | AF027706 |
|  | AAC34970 |
|  | AF064824 |
|  | AAC25668 |
|  | AF078530 |
|  | AAC27722 |
|  | AI824070 |
|  | AK075213 |
|  | BAG52085 |
|  | AK301448 |
|  | BAH13484 |
|  | AY358813 |
|  | AAQ89172 |
|  | AY358814 |
|  | AAQ89173 |
|  | AY524045 |
|  | AAS94254 |
|  | AY562996 |
|  | AAS75586 |
|  | BC004553 |
|  | AAH04553 |
|  | DA040898 |
|  | KU178437 |
|  | ALQ33895 |
|  | KU178438 |
|  | ALQ33896 |
|  | O43353 |
|  | NC\_000070 |
|  | XM\_006537677 |
|  | XP\_006537740 |
|  | XM\_006537679 |
|  | XP\_006537742 |
|  | XR\_390315 |
|  | XM\_006537678 |
|  | XP\_006537741 |
|  | NM\_138952 |
|  | NP\_620402 |
|  | NM\_001329751 |
|  | NR\_138102 |
|  | AL807379 |
|  | CH466538 |
|  | EDL05589 |
|  | EDL05590 |
|  | AF461040 |
|  | AAL96436 |
|  | AF487539 |
|  | AAM76919 |
|  | AK159593 |
|  | BAE35213 |
|  | AK172319 |
|  | BAE42942 |
|  | AK172417 |
|  | BAE42997 |
|  | AK192973 |
|  | AK217339 |
|  | BC050845 |
|  | BC058088 |
|  | BC069878 |
|  | AAH69878 |
|  | P58801 |
|  | NC\_005104 |
|  | XM\_017593470 |
|  | XP\_017448959 |
|  | NM\_001191865 |
|  | NP\_001178794 |
|  | AC\_000073 |
|  | AABR07047366 |
|  | AAHX01035130 |
|  | AAHX01035131 |
|  | CH473962 |
|  | EDL98485 |
|  | BC107468 |
|  | AAI07469 |
|  | FQ230093 |
|  | NC\_018919 |
|  | AMYH02018389 |
|  | AC\_000026 |
|  | AAHY01033792 |
|  | AAHY01033793 |
|  | AAHY01033794 |
|  | BC025611 |
|  | AAH25611 |
|  | AC\_000140 |
|  | CAM22557 |
|  | NT\_008046 |
|  | NW\_001839136 |
|  | NW\_923984 |
|  | AC\_000051 |
|  | CR592019 |
|  | CR596906 |
|  | Q2TU65 |
|  | Q6Q322 |
|  | NT\_039258 |
|  | NW\_001030740 |
|  | Q547H1 |
|  | XM\_342810 |
|  | XP\_342811 |
|  | NW\_047711 |
|  | NW\_001084836 |
|  | XM\_001056736 |
|  | XP\_001056736 |
|  | Q3B7U0 |
|  | ABBA01037399 |
|  | ABBA01037400 |

---

|  |  |
| --- | --- |
| Swiss-Prot ID | RIPK2\_HUMAN |
|  | RIPK2\_MOUSE |
|  | Q547H1\_MOUSE |

---

|  |  |
| --- | --- |
| Cell Localization | Cytoplasm |

---

|  |  |
| --- | --- |
| Ensembl ID | ENSG00000104312 |
|  | ENSP00000220751.4 |
|  | ENST00000220751.5 |
|  | ENSMUSG00000041135 |
|  | ENSMUSP00000038833.5 |
|  | ENSMUST00000037035.11 |
|  | ENSMUST00000183871.1 |
|  | ENSRNOG00000009389 |
|  | ENSRNOP00000012963.2 |
|  | ENSRNOT00000012963.5 |
|  | ENSP00000220751 |
|  | ENST00000220751 |
|  | ENSMUSP00000038833 |
|  | ENSMUST00000037035 |
|  | ENSMUST00000183871 |
|  | ENSRNOP00000012963 |
|  | ENSRNOT00000012963 |

---

|  |  |
| --- | --- |
| MGI ID | MGI:1891456 |
|  | 1891456 |

---

|  |  |
| --- | --- |
| RGD ID | 1309167 |

---

|  |  |
| --- | --- |
| Unigene ID | Hs.103755 |
|  | Mm.112765 |
|  | Rn.102179 |

---

|  |  |
| --- | --- |
| Homologene ID | 37856 |

---

|  |  |
| --- | --- |
| Shape | Sickle-vertex |

---

|  |  |
| --- | --- |
| IPI ID | IPI00139382 |
|  | IPI00021917 |
|  | IPI00554774 |
|  | IPI00816174 |
|  | IPI00207880 |
|  | IPI00766118 |

---
